# Electrochemical Response of *Saccharomyces cerevisiae* Corresponds to Cell Viability upon Exposure to *Dioclea reflexa* Seed Extracts and Antifungal Drugs

**DOI:** 10.3390/bios9010045

**Published:** 2019-03-20

**Authors:** Patrick Kobina Arthur, Anthony Boadi Yeboah, Ibrahim Issah, Srinivasan Balapangu, Samuel K. Kwofie, Bernard O. Asimeng, E. Johan Foster, Elvis K. Tiburu

**Affiliations:** 1 Department of Biochemistry, Cell and Molecular Biology, University of Ghana, Legon P.O. Box LG 54, Ghana; parthur14@gmail.com; 2 West African Centre for Cell Biology of Infectious Pathogens, University of Ghana, Legon P.O. Box LG 54, Ghana; srinivasan_bs85@yahoo.com (S.B.); skwofie2000@gmail.com (S.K.K.); 3 Department of Biomedical Engineering, School of Engineering Sciences, College of Basic and Applied Sciences, University of Ghana, Legon P.O. Box LG 25, Ghana; ayboadi@st.ug.edu.gh (A.B.Y.); issahi62@gmail.com (I.I.); boasimeng@ug.edu.gh (B.O.A.); 4 Department of Medicine, Loyola University Medical Center, Chicago, IL 60153, USA; 5 Department of Materials Science and Engineering, Virginia Tech, Blacksburg, VA 24061, USA; johanf@vt.edu

**Keywords:** electrochemical detection, *Dioclea reflexa*, bioactive, amphotericin, rifampicin, cell viability

## Abstract

*Dioclea reflexa* bioactive compounds have been shown to contain antioxidant properties. The extracts from the same plant are used in traditional medical practices to treat various diseases with impressive outcomes. In this study, ionic mobility in *Saccharomyces cerevisiae* cells in the presence of *D. reflexa* seed extracts was monitored using electrochemical detection methods to link cell death to ionic imbalance. Cells treated with ethanol, methanol, and water extracts were studied using cyclic voltammetry and cell counting to correlate electrochemical behavior and cell viability, respectively. The results were compared with cells treated with pore-forming Amphotericin b (Amp b), as well as Fluconazole (Flu) and the antimicrobial drug Rifampicin (Rif). The *D. reflexa* seed water extract (SWE) revealed higher anodic peak current with 58% cell death. Seed methanol extract (SME) and seed ethanol extract (SEE) recorded 31% and 22% cell death, respectively. Among the three control drugs, Flu revealed the highest cell death of about 64%, whereas Amp b and Rif exhibited cell deaths of 35% and 16%, respectively, after 8 h of cell growth. It was observed that similar to SWE, there was an increase in the anodic peak current in the presence of different concentrations of Amp b, which also correlated with enhanced cell death. It was concluded from this observation that Amp b and SWE might follow similar mechanisms to inhibit cell growth. Thus, the individual bioactive compounds from the water extracts of *D. reflexa* seeds could further be purified and tested to validate their potential therapeutic application. The strategy to link electrochemical behavior to biochemical responses could be a simple, fast, and robust screening technique for new drug targets and to understand the mechanism of action of such drugs against disease models.

## 1. Introduction

Electrochemical detection of drugs that interact with most biological systems is an important strategy to understand cellular stresses that cause cell death [1,2,3]. *Saccharomyces cerevisiae* (*S. cerevisiae*) shares the complex internal cell structure of animal cells and serves as an ideal model for conducting research in higher eukaryotes. *S. cerevisiae* has been used extensively to study cellular mechanisms, including DNA damage and repair as well as systematic fungal infections [4,5,6]. Evidence from previous findings indicate that there are several membrane redox centers in most eukaryotic cells that can be targeted to monitor redox activities in the presence of certain drugs [7,8,9]. 

There are essentially two pathways (lipid-mediated and diffusion porins) through which both hydrophobic and hydrophilic antimicrobials elicit their potency. The degree of permeation of the cell membrane has a major impact on the redox activity. In addition, the presence of a hydrophobic drug within the complex architecture of the membrane induces pore formation and enhances ionic flow, which can be detected electrochemically. Non-membrane-mediated drugs diffuse freely through the membrane and may not necessarily destabilize the membrane architecture; therefore, the ionic flow that can be captured by electrochemical detection techniques is limited.

The construction and maintenance of a high-quality natural products library based on microbial, plant, marine, or other sources is a resource-intensive endeavor. Crude extract libraries have lower resource requirements for sample preparation and allow for rapid screening of bioactive compounds. Until now, the mechanistic studies of natural products through high throughput screening (HTS) requires high quality natural product library [10,11]. While HTS provide a thorough understanding of drug behavior that can inform further characterization, the procedures are also resource-intensive. Screening methods, especially for antimicrobial lead compounds, have been a major challenge, and a number of modifications to the methods have been made over the years [12]. We therefore intend to develop a simple procedure based on electrochemistry that allows simple and rapid screening of membrane-targeted leads. The concept is based on the premises that membrane modulation of a ligand can offset the chemical balance, thereby enhancing the flow of ions/charges and potentiating the activities of antimicrobial compounds [6,13]. To test these hypotheses, *Dioclea reflexa* seed extracts and three extensively studied antifungal and antibiotic drugs were selected to investigate their electrochemical behaviors using *S. cerevisiae* cells as a model biological system. 

*D. reflexa* is a leguminous plant that is commonly found in tropical Africa and South America. Previous studies on *D. reflexa* revealed remarkable medicinal properties, including antioxidant and inflammation activities, which have been exploited to treat a number of diseases with extremely impressive outcomes [14,15]. The leaves and seeds of the plant have phytochemical compounds, which possess antimicrobial and antioxidant properties, and are used to treat typhoid, asthma, and rheumatism [13,14,15]. However, the detailed mechanism of action of this plant extracts in treating almost all the diseases mentioned is not fully understood. In this work, ethanol, methanol, and water extracts of the bioactive molecules in *D. reflexa* seeds will be used to study the electrochemical behavior of *S. cerevisiae* cell lines, and the results are correlated to cell death for identifying potential drug leads [16].

One of the most extensively used antimicrobial drugs for studying *S. cerevisiae* is amphotericin b (Amp b) and fluconazole (Flu), which is used as a fungistatic drug [17,18,19]. Another drug, rifampicin (Rif), which is a strong antibiotic against tuberculosis (TB), has also been extensively studied in model TB strains [20]. Amp b, Flu, and Rif are used as controls. Amp b is a membrane-mediated drug that increases the permeability of ions and small molecules by binding to ergosterol in the *S. cerevisiae* membrane to create pores [21,22]. Flu, on the other hand, has an antifungal influence on *Candida albicans* as well as other fungal diseases because it inhibits ergosterol synthesis, whereas Rif binds to the beta subunit of the DNA-dependent RNA polymerase enzyme complex to inhibit the transcription of messenger RNA in TB strains. Unlike Amp b and Flu, Rif does not exhibit antifungal activity, but it can diffuse freely through different organelles. Thus, the electrochemical response of the cells in the presence of the three extracts will be compared to cells treated with these commercial drugs to validate the feasibility of such techniques in screening plant products. The aim of this work is to develop ion channel mimetic biosensors for detecting membrane-targeted natural products using amperometric response mechanisms.

## 2. Methods

### 2.1. Growth Medium and Cell Culturing

A cell culture of *S. cerevisiae* (ATCC/LGC Standards, Teddington, Middlesex, UK) was maintained on YEPD agar at 4 °C (Yeast extract Peptone, Dextrose, and granulated Agar). Yeast cultures were grown in 150 mL of YEPD broth in shake flasks rotated at 180 rpm for 16 h at 30 °C. The cells were then harvested by centrifugation at 16,000 rpm and washed twice in 25 mL of 50 mM phosphate buffer of pH 7. The cells were then re-suspended in sterile phosphate-buffered saline (PBS, 50 mM K_2_HPO_4_/KH_2_PO_4_, pH 7, 100 mM KCl) (Sigma-Aldrich, St. Louis, MO, USA). The optical density of the cell suspension was adjusted to give an OD_600_ of 40 using an LKB Novaspec 11 spectrophotometer (Pharmacia Biotech, Piscataway, NJ, USA). The cells were used on the day of harvest at a seeding density of 2.15 × 10^3^ cells/cm^2^.

### 2.2. Seed Drying and Extraction and Drug Acquisition

The fresh seeds of *Dioclea reflexa* were obtained from a farm in Suma Ahenkro in the District of Jaman North in the Brong Ahafo region of Ghana (coordinates: 7°57′1.8″ North and 2°41′52.08″ West). The seeds were identified by Prof. Isaac Kojo Asante, the head of the Department of Botany at the University of Ghana. The cotyledon inside the pericarp was dried for ten days in the open sun, after which the seed was cracked open and dried for an additional ten days under room temperature. When the seed was fully dried, the cotyledon was ground to a powder using a laboratory mortar and pestle. All commercial drugs were obtained from a vendor (Sigma-Aldrich, Saint Louis, MO, USA).

### 2.3. Solvent Extraction

Five grams of the seed powder was mixed with 30 mL of each solvent (70% ethanol, 70% methanol, and 100% deionized water, all solvents were obtained from Sigma-Aldrich, St. Louis, MO, USA with 99.9% purity). The mixture was then rotated on an orbital mixer for 48 h. It was later removed and then allowed to settle. The supernatants from all the extractions were freeze-dried, and the resulting powder was reconstituted with 1000 µL of 70% ethanol. UV-VIS absorption measurements were done using a JENWAY, 6705 UV-Vis Spectrophotometer (Cole-Parmer, Staffordshire, UK).

### 2.4. Cell Viability Measurements Using Trypan Blue Based Assay 

A stock solution of 1 mg/mL of the drug or the extracts were prepared separately using dimethyl sulfoxide (≥99.7% purity) (Sigma-Aldrich, St. Louis, MO, USA). The final drug or extract concentration in 200 µL of cells ranges from 5–30 µg/mL [23]. The cells were incubated with the drugs or the extracts in time intervals ranging from 20 min to 8 h. Twenty microliters of cells were added to 20 µL of 0.2% trypan blue, prepared in PBS at pH = 7.2 and mixed thoroughly. After which 20 µL of the resulting solution was pipetted and then deposited onto the counting chamber for the cell viability studies using a Nexcelom Cellometer (Nexcelom Bioscience, Lawrence, MA, USA).

Electrochemical detection of the cells was done using cyclic voltammetry under steady-state conditions. A CheapStat potentiostat device (IO Rodeo, Pasadena, CA, USA) was used in all experiments. Interdigitated Gold Electrodes (IDEs)/Microelectrodes was purchased from Metrohm, DropSens (Llanero, Spain) and composed of two interdigitated electrodes with two connection tracks all made of gold on a glass substrate. The design of the Interdigitated electrodes allows two electrodes to fuse together, and as a result, the distance between two electrodes is reduced. The electrodes were thoroughly cleaned and polished before each measurement. The potentiostat was held at open circuit prior to each scan, and the cyclic voltammograms were obtained by scanning from 690 mV to 970 mV at a scan rate of 10 mV/s. Notably, the position of the voltammogram on the current axis gave an immediate indication of the proportions of each quantification of the redox form [17]. 

## 3. Results 

### 3.1. Structure of the Antifungal Drugs and Schematic of the Study

**Figure 1:**
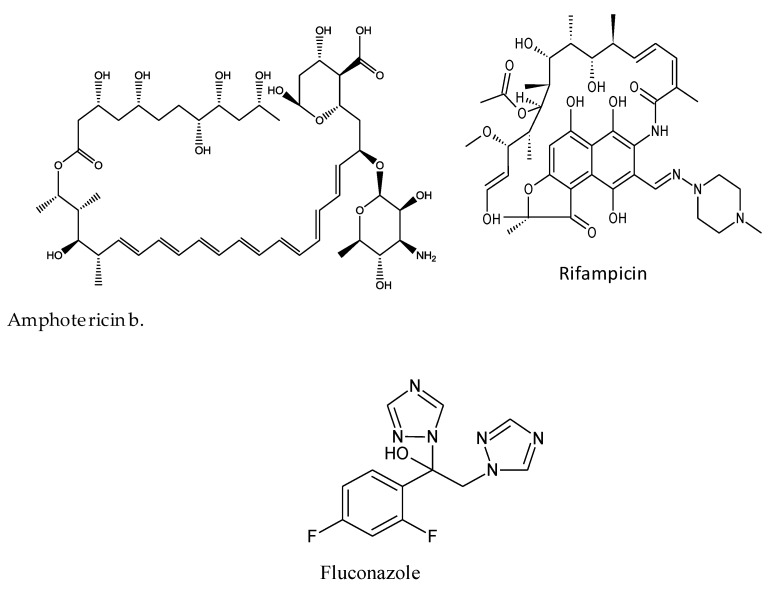
The chemical structures of amphotericin b (Amp b), fluconazole (Flu), and rifampicin (Rif). As depicted, each of these drugs have unique structural features that can influence membrane integrity.

**Figure 2:**
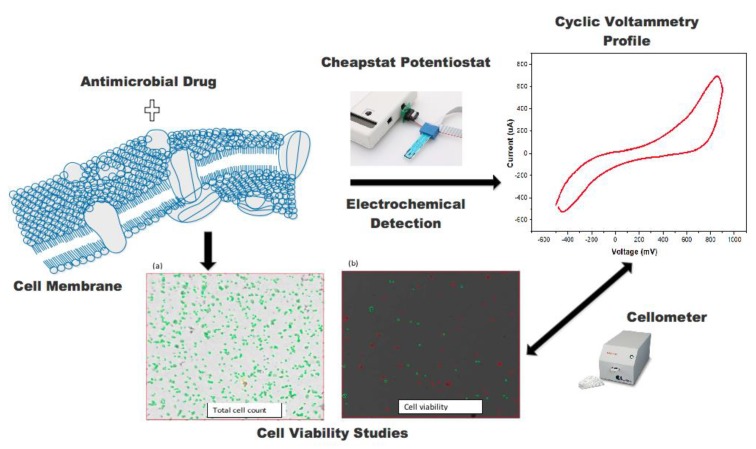
Schematic illustration of the mechanism of drug interaction with biological membranes and how its electrochemical response (using a miniature electrode) correlates to cell viability, as captured by the cell counting device (Cellometer).

The chemical structures of amphotericin (Amp b), fluconazole (Flu), and rifampicin (Rif) are shown in Figure 1. Amp b is a polyene with seven adjoining trans double bonds. Flu is a synthetic triazole with fungistatic activity, whereas Rif is a semisynthetic antibiotic obtained from *Streptomyces*. The stepwise procedure in this work was to probe the mechanism of action of the antimicrobial drugs and plant extracts, as shown in Figure 2. First, the drug/constituents of the plant extracts were used to target the membrane environment and cause membrane depolarization, leading to the formation of pores with an increased permeability to protons and monovalent ions such as Na^+^ and K^+^. The ionic transfer was captured through electrochemical detection followed by cell viability measurements to determine the correlation between ionic mobility across the biological membrane and cell death.

### 3.2. UV-VIS Spectrophotometry Studies

**Figure 3:**
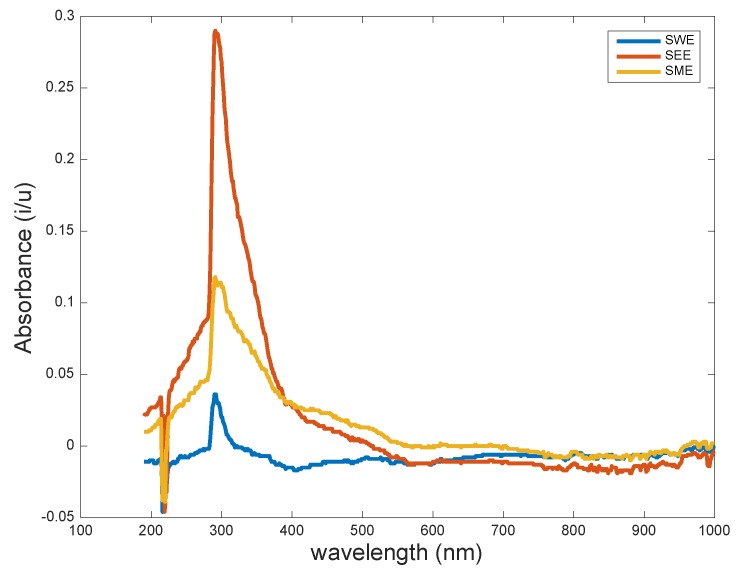
UV-VIS profile for the seed water extract (SWE), seed ethanol extract (SEE), and seed methanol extract (SME) revealed intense peaks all centered around 293 nm. The SWE also indicated the least peak intensity, implying a low extraction efficiency with water.

The UV/VIS monitoring of the extracts showed that SEE and SME were very efficient in removing the bioactive compounds, whereas SWE revealed the least as shown in Figure 3. As expected, the lower absorbance value from the water extracts was presumably due to the fact that either most of the hydrophobic compounds could not be extracted into the aqueous phase, or the bioactive compounds were not UV/VIS active. Methanol and ethanol, however, were more efficient in extracting the bioactive molecules resulting in higher peak absorbance intensities. Nonetheless, the major UV/VIS absorbance wavelengths were in the same range (290–293 nm) for all the extracts and confirmed previous studies of *D. reflexa*—that extracts had unique wavelength characteristics in the presence of antioxidants, phenolic compounds, alkaloids, flavonoids, cinnamaldehydes, benzene, and lignin derivatives [14,23,24]. 

### 3.3. Cyclic Voltammetry and Cell Viability Studies of S. cerevisiae Cells Treated with Extracts

**Figure 4:**
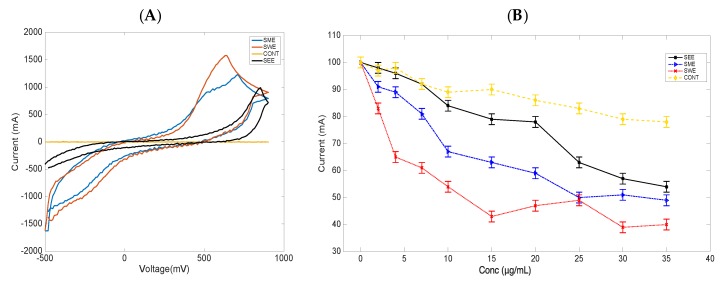
(**A**) Cyclic Voltammetry profile of the extracts without cells showing characteristic anodic signals of the main natural products in the seed water extract (SWE, red), seed methanol extract (SME, blue), and seed ethanol extract (SEE, black), compared with the bare electrode (CONT, yellow). (**B**) Concentration-dependent cell viability studies of the extracts on *S. cerevisiae* cell lines: SWE, red; SME, blue; and SEE, black, compared with the bare electrode, CONT, yellow. Reproducibility of the data was analyzed using triplicate measurements.

To test the electrochemical behavior and redox activity of the extracts, cyclic voltammetry analysis was conducted using interdigitated gold electrodes (IDEs), (Metrohm, DropSens). Briefly, the IDEs were composed of two interdigitated electrodes with two connection tracks on a glass substrate and offered several advantages, such as working with low volumes of samples and avoiding tedious polishing of solid electrodes. There were no redox peaks observed from the bare electrodes, as shown in Figure 4A (CONT, yellow), however, all the extracts showed quasi-reversible oxidation processes in 0.1% DMSO with current values that ranged from 0.10 to 0.18 mA at a scan rate of 10 mV/s, as shown in Figure 4A for SWE, red; SME, blue; and SEE, black. The SWE exhibited a higher oxidative peak current, which was shifted to the left, probably indicating that most of the bioactive compounds were oxidative species compared to those in the SEE and SME extracts. The corresponding concentration-dependent cell viability studies were conducted for each extract, and the results are shown in Figure 4B. It was observed that SWE (red) demonstrated the most cell death, followed by SME (blue) and SEE (black), and the untreated cells CONT (yellow) exhibited the least cell viability with concentrations up to 30 µg/mL and an incubation time of 8 h. It was noted that prolonging incubation beyond 8 h resulted in programmed cell death, and the cell counter continually indicated error messages.

**Figure 5:**
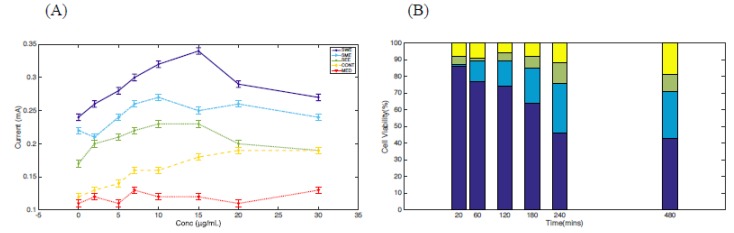
(**A**) Concentration-dependent profile of the change in anodic current as a function of extract concentration after 8 h of extract administration (SWE, purple; SME, blue; and SEE, green) compared to untreated cells (CONT, yellow) and the medium in which the cells were cultured (MED, red). (**B**) Percent cell viability at 15µg/mL extract concentration with data recorded from 20 min to 8 h using trypan blue as a staining dye. SWE, purple; SME, blue; and SEE, green were compared to the untreated cells (CONT, yellow) after 8 h. Reproducibility of the data was analyzed using triplicate measurements.

When each extract was tested on *S. cerevisiae* cell lines, with similar concentrations ranging from 5 to 30 µg/mL, distinct anodic peak currents were recorded at 15 µg/mL for the SWE extract (purple), as shown in Figure 5A. Also, a noticeable redox activity was observed in the presence of SME (blue) and SEE (green) compared to the untreated cells (CONT, yellow) and the medium in which the cells were cultured (MED, red). The metabolites in the media also recorded modest peak currents in the same concentration range as shown in Figure 5A (MED, red). The results were interpreted in terms of one or more biological processes including increased biological membrane porosity in the presence of the extracts with SWE, exhibiting the most influx of ions at a 15 µg/mL extract concentration or the release of reactive oxygen species (ROS) as a result of the presence of the extracts. We also correlated ionic leakage to cell death by conducting cell viability studies with an extract concentration of 15 µg/mL and with cells incubated for 8 h, as shown in Figure 5B. SWE (purple) revealed cell death of about 57%, whereas SME (blue) and SEE (green) recorded about 31% and 22%, respectively, at the same concentration. Cell death was recorded in an increasing order, SEE < SME < SWE, which correlated with the oxidative peak current in the same order, suggesting that electrochemical responses from the cells might have resulted in cellular stress, leading to the highest cell death in the presence of the *D. reflexa* cell extract (especially SWE). 

### 3.4. Cyclic Voltammetry Studies of S. cerevisiae in the Presence of Antifungal Drugs

**Figure 6:**
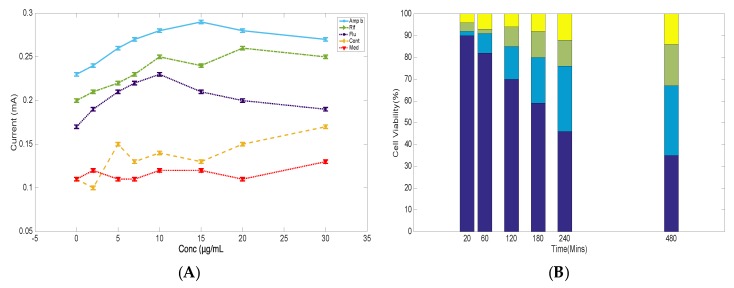
(**A**) Concentration-dependent profile of the change in anodic current as a function of drug concentration (amp b, blue; Rif, green; Flu, purple; untreated cells, CONT, yellow; and medium, MED, red) after 8 h of drug administration. (**B**) Percent cell viability at 15 µg/mL extract concentration with data recorded from 20 min to 8 h using trypan blue as a staining dye. Reproducibility of the data was analyzed using triplicate measurements.

The extract data was compared to Amp b because the antifungal ability of the drug to cause cell death has been linked to cellular stress and ionic leakage, with numerous electrochemical studies suggesting a correlation between membrane permeability of ions to the drug mechanism of action [24,25]. As shown in Figure 6A, cyclic voltammetry measurements of treated cells with drugs were compared to the cells alone. Amp b (blue) treated cells with concentrations ranging from 5 to 30 µg/mL showed elevated anodic responses, indicating ionic leakage from a porous membrane, as previously demonstrated [24,25]. Similar treatment with Flu (purple) showed no dramatic changes in anodic peak current in the presence of the drug, and this observation was the same when the cells were treated with Rif (green) after eight hours of drug exposure, compared to the Amp b current response within the margin of statistical error. Prior to the treatment of the cells with the drugs, cyclic voltammetry analysis indicated that Amp B was not redox active, yet its mechanism of action could create pores in the cellular membrane to enhance the influx of ions in and out of the membrane, indicating membrane-mediated effects (data not shown). Thus, it was confirmed that Flu and Rif were not membrane-medicated, and, therefore, membrane permeation of ions was very minimal as revealed in the data. These observations attested to earlier studies that the mechanism of action of these drugs was through different pathways, but they could still demonstrate some level of electrochemical responses [26,27]. 

The second phase was to correlate the electrochemical response of each drug to cell viability, as shown in Figure 6B. The cell viability percentage was recorded using trypan blue as the staining agent from 20 min to 8 h in the presence of each drug, as was performed with the extracts. The cell viability results after 20 min did not show a drastic change in cell death when treated with the drugs. However, there was a drastic change in cell viability of about 64% in the presence of Flu (purple) after eight hours. Amp b (blue) showed cell death of about 35%, whereas Rif (green) recorded about 16% cell death at concentrations of 15 µg/mL. Previous studies on Flu indicated that cell death was not membrane-mediated, and was, therefore, used as a control fungistatic drug, whereas Rif served as an antibacterial drug that has no antifungal properties. It was, therefore, concluded from this study that alterations of the endogenous membrane due to the presence of Amp b might have resulted in an enhanced influx of ions, and this correlated to the increased cell death that has already been documented [22,28]. As previous findings have indicated, many biological membranes have electrochemical characteristics, which is important for the generation of electron transfer in living systems [29,30,31,32]. Thus, endogenous chemical imbalances and an increased influx of drugs in the cell can result in cellular stress, leading to cell death. Any new molecule or drug entity with the ability to increase membrane permeability of ions is likely to increase cellular stress. While it is acknowledged that cell death may show different mechanism of inhibition, it is expected from this study that plant extracts, or isolated bioactive compounds from plants, can be used to study cellular stress and correlate their activity to cell death using both electrochemical and cell viability studies [33,34]. 

### 3.5. Discussion

There are numerous redox mediators that are used to either study cell redox activity or develop biosensors for many biological systems. For example, *S. cerevisiae* has several redox centers, such as [Fe(CN)_6_]^3−^/[Fe(CN)_6_]^4−^ and NAD(P)H/NAD(P)^+^, which can be targeted by hydrophilic/hydrophobic molecules, as extensively discussed previously by Rawson et al. [35,36]. In addition, electrochemical monitoring can be conducted when reactive oxygen species are released as a result of drug-induced action [32,37,38,39,40]. Reactive oxygen species is a term used to describe oxygen species, including superoxide anion radical (O_2_^•−^) and hydrogen peroxide (H_2_O_2_), and they can cause cytotoxic and antimicrobial effects in most organisms. Thus, there are at least three main mechanisms of drug action including, but not limited to, membrane depolarization leading to an influx of ions (as in the case of Amp b), targeting of redox centers, and/or the release of ROS in *S. cerevisiae* [28,35,36,41]. Amp b has been used to treat fungal infection, and the mechanism through which the antifungal drug kills infected cells is well-characterized using both biophysical and microbiological techniques [22,25]. The antifungal drug binds to ergosterol and forms pores at the cell membrane, causing the loss of ions and leading to depolarization of the membrane [13,21]. This mechanism can cause an enhanced oxidation potential, which can be captured through electrochemical detection, as already observed in our studies as well as studies from other groups [25,36]. 

In the case of the two other mechanisms, an enhanced anodic response can result from either targeting redox mediator centers or generating one or more ROS species as a result of drug binding, as depicted below:

(1) $$\mathrm{NADPH}\;\underset{H^{+}\;+\;{2e}^{-}}{↔}\;{\mathrm{NADP}}^{+},$$

H_2_O_2_ ⟶ O_2_ (*g*) + 2H^+^ (*aq*) + 2e^−^. (2)

Whereas the reduction peaks seemed to deviate from a typical redox reaction, the research suggested that the effect of Amp b (35%) or the plant extracts (57%) on *S. cerevisiae* viability, which corresponded to the quasi-reversible oxidation process, inevitably supported a general claim that Amp b (57%) and the plant extracts (SWE, SME, SEE at 57%, 31%, and 22%, respectively) behave similarly and have the ability to create ionic pores in fungal cells that leads to their death. Our data was reproducible because it used triplicate measurements from the same extract or from different extracts of the same plant, with error margins of less than 5% confidence. The selectivity towards the extracts was validated using the non-membrane-targeted antimicrobial drug, Rif, with the extracts, revealing up to 57% antifungal activity compared to 16% of the antimicrobial drug. These studies provide a sensitive sensing method that can electrochemically detect plant extracts, causing either depolarization of membranes, accumulation of ROS, or targeting redox centers in *S. cerevisiae* cell cultures. Whereas the fabrication of microelectrodes for electrochemical studies has been going on for one or two decades in various fields, there is limited application in the natural product field. The limited evidence of the application of electrochemical methods in natural products screening, as shown in Table 1, might be due to the high cost of operation [42]. As shown in Table 1, our method offers a simple and straightforward screening platform to select active natural product pools in a fast and robust fashion prior to undertaking further validation studies [43,44].

## 4. Conclusions

At a concentration of 15 µg/mL, *S. cerevisiae* cell lines revealed unique oxidation peak responses at 0.34, 0.25, and 0.23 in the presence of SWE, SME, and SEE of the *D. reflexa* extracts, respectively, correlating to cell death of 57%, 31%, and 22% in that order. The results were comparable to Amp b on *S. cerevisiae* cell death, where the highest oxidation peak current was directly related to inhibitory effects. Thus, one or many bioactive compounds in *D. reflexa* might induce cell death through similar mechanisms as Amp b. Future work will focus on the individual bioactive compounds from the water extracts of *D. reflexa* seeds to validate their potential therapeutic application. In conclusion, the current studies have highlighted the robustness of electrochemical detection in monitoring cell death using microliter volumes of sample. 

## Figures and Tables

**Table 1:** Comparing the various methods for screening natural product activity towards molecular targets.

Method	Strategy	Sample Size	Robustness	Incubation Time (h)	Disadvantages
The current Method	Uses Interdigitated electrode and Cellometer	microliters	Fast and Sensitive	0.3–8	Preliminary Target not known
Fluorescence techniques	Based on staining and counter staining	milliliters	Complex architecture	long	phototoxic-unclear images [44]
High Performance Liquid Chromatography-Electrochemical Detection (HPLC-ECD)	A separation technique	microliters	selective and sensitive	Relatively fast	Expensive [44]
Structural Activity Relationship by NMR (SAR by NMR)	nuclear magnetic resonance (NMR)-based	microliters	target-directed drug research	Short	Focuses on hit validation studies [43]
Diffusion Methods	Agar plates are inoculated and compared to standard	large	Simplicity and low cost	16–24	Inaccuracies [43]
